# Bis(ethano­lato-κ*O*)(5,10,15,20-tetra­phenyl­calix[4]pyrrole)manganese(III) hexa­fluoro­phosphate

**DOI:** 10.1107/S1600536808021879

**Published:** 2008-09-06

**Authors:** Suwen Wang, Zhongfang Li, Xutao Wang, Xianjin Yu

**Affiliations:** a College of Chemical Engineering, Shandong University of Technology, Zibo 255049, People’s Republic of China

## Abstract

The title compound, [Mn(C_2_H_5_O)_2_(C_44_H_28_N_4_)]PF_6_, was synthesized from manganese(III) 2,4-penta­nedionate and 5,10,15,20-tetra­phenyl­calix[4]pyrrole by a hydro­thermal reaction. The Mn^III^ atom is located on an inversion centre and the asymmetric unit comprises one half-formula unit. The Mn^III^ ion is hexa­coordinated by four N atoms from one 5,10,15,20-tetra­phenyl­calix[4]pyrrole ligand and two O atoms from two deprotonated ethanol mol­ecules. The equatorially located atoms (the Mn and four N atoms) are planar. The dihedral angles between the planes of the phenyl rings and the equatorial plane are 53.3 (2) and 81.8 (2)°. One hexa­fluoro­phosphate anion balances the charge.

## Related literature

For related literature, see: Church & Halvorson (1959 ▶); Chung *et al.* (1971 ▶); Okabe & Oya (2000 ▶); Serre *et al.* (2005 ▶); Pocker & Fong (1980 ▶); Scapin *et al.* (1997 ▶). 
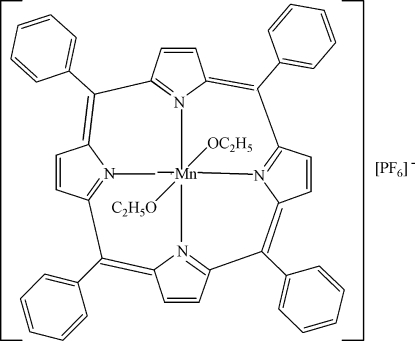


## Experimental

### 

#### Crystal data

[Mn(C_2_H_5_O)_2_(C_44_H_28_N_4_)]PF_6_
*M* *_r_* = 902.73
Monoclinic, 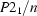

*a* = 10.7487 (8) Å
*b* = 16.8682 (14) Å
*c* = 11.9913 (19) Å
β = 109.412 (9)°
*V* = 2050.6 (4) Å^3^
*Z* = 2
Mo *K*α radiation
μ = 0.43 mm^−1^
*T* = 293 (2) K
0.43 × 0.28 × 0.22 mm

#### Data collection

Bruker APEXII CCD area-detector diffractometer
Absorption correction: multi-scan (*SADABS*; Bruker, 2001 ▶) *T*_min_ = 0.835, *T*_max_ = 0.910
4407 measured reflections
3535 independent reflections
2142 reflections with *I* > 2σ(*I*)
*R*_int_ = 0.040

#### Refinement

*R*[*F* ^2^ > 2σ(*F* ^2^)] = 0.063
*wR*(*F* ^2^) = 0.214
*S* = 1.00
3535 reflections
284 parameters
H-atom parameters constrained
Δρ_max_ = 0.33 e Å^−3^
Δρ_min_ = −0.42 e Å^−3^

### 

Data collection: *APEX2* (Bruker, 2004 ▶); cell refinement: *SAINT-Plus* (Bruker, 2001 ▶); data reduction: *SAINT-Plus*; program(s) used to solve structure: *SHELXS97* (Sheldrick, 2008 ▶); program(s) used to refine structure: *SHELXL97* (Sheldrick, 2008 ▶); molecular graphics: *SHELXTL* (Sheldrick, 2008 ▶); software used to prepare material for publication: *SHELXTL*.

## Supplementary Material

Crystal structure: contains datablocks global, I. DOI: 10.1107/S1600536808021879/kp2179sup1.cif

Structure factors: contains datablocks I. DOI: 10.1107/S1600536808021879/kp2179Isup2.hkl

Additional supplementary materials:  crystallographic information; 3D view; checkCIF report

## Figures and Tables

**Table:** 

Mn1—N1	2.004 (3)
Mn1—N2	2.018 (3)
Mn1—O1	2.260 (3)

**Table:** 

N1—Mn1—N2	90.13 (13)
N1—Mn1—O1^i^	90.70 (13)
N2—Mn1—O1^i^	90.25 (13)
N2—Mn1—O1	89.75 (13)

Symmetry code: (i) 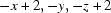.

**Fig. 1.**
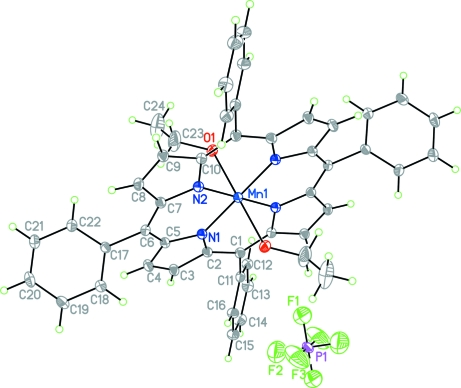
The molecular structure of (I) drawn with the 30% probability displacement ellipsoids for the non-hydrogen atoms. Symmetry codes used: - x + 2,-y,-z + 2; - x +1,-y,-z).

## supplementary crystallographic information

### Comment

In recent years, nitrogen-containing organics have been widely used as polydentate ligands, which can coordinate to transition or rare earth ions yielding complexes with interesting properties that are useful in materials science (Church & Halvorson, 1959; Chung *et al.*, 1971) and in biological systems (Okabe & Oya, 2000; Serre *et al.*, 2005; Pocker & Fong, 1980; Scapin *et al.*, 1997). Herein, we report the synthesis and X-ray crystal structure analysis of the title compound, {[5,10,15,20-tetraphenyl-calix[4]pyrrole]manganese(III) bis-ethanol} hexafluorophosphate.

The Mn^III^ ion is hexa-coordinated with four N atoms from one 5,10,15,20-tetraphenyl-calix[4]pyrrole ligand and two O atoms from two ethanol molecules (Fig. 1). One hexafluorophosphate anion acts as charge balance. Mn^III^ is located at the inversion centre and the asymmetric unit comprises one-half molecule. The Mn^III^ ion is hexa-coordinated with four N atoms from one 5,10,15,20-tetraphenyl-calix[4]pyrrole ligand and two O atoms from two ethanol molecules. The equatorially located atoms  Mn(1), N(1), N(2), N(1 A), and N(2 A)  are  planar. The dihedral angles between the planes of Ph rings [C(11), C(12), C(13), C(14), C(15), C(16)] and [C(17), C(18), C(19), C(20), C(21), C(22)] and quatorial plane are 53.3 (2) and 81.8 (2) Å, respectively. The Mn—N and Mn—O bond lengths are in the range of 2.004 (3)–2.018 (3) and 2.260 (3) Å, respectively (Table 1).

### Experimental

A mixture of manganese(III) 2,4-pentanedionate (0.5 mmol), 5,10,15,20-tetraphenyl-calix[4]pyrrole (0.5 mmol), H_2_O (8 ml) and ethanol (8 mL) in a 25 mL Teflon-lined stainless steel autoclave was kept at 433 K for three days. Red crystals were obtained after cooling to room temperature with a yield of 18%. Anal. Calc. for C_48_H_38_MnN_4_O_2_F_6_P: C 66.61, H 3.82, N 6.48%; Found: C 66.65, H 3.78, N 6.52%.

### Refinement

All H atoms were placed in calculated positions with C—H = 0.93Å and refined as riding with *U*_iso_(H) = 1.2*U*_eq_(carrier).

### Crystal data

**Table:**

[Mn(C_2_H_5_O)_2_(C_44_H_28_N_4_)]PF_6_	*F*(000) = 928
*M**_r_* = 902.73	*D*_x_ = 1.462 Mg m^−^^3^
Monoclinic, *P*2_1_/*n*	Mo *K*α radiation, λ = 0.71073 Å
Hall symbol: -P 2yn	Cell parameters from 3535 reflections
*a* = 10.7487 (8) Å	θ = 2.2–25.0°
*b* = 16.8682 (14) Å	µ = 0.44 mm^−^^1^
*c* = 11.9913 (19) Å	*T* = 293 K
β = 109.412 (9)°	Block, yellow
*V* = 2050.6 (4) Å^3^	0.43 × 0.28 × 0.22 mm
*Z* = 2	

### Data collection

**Table:**

Bruker APEXII CCD area-detector diffractometer	3535 independent reflections
Radiation source: fine-focus sealed tube	2142 reflections with *I* > 2σ(*I*)
graphite	*R*_int_ = 0.040
φ and ω scans	θ_max_ = 25.0°, θ_min_ = 2.2°
Absorption correction: multi-scan (*SADABS*; Bruker, 2001)	*h* = −1→12
*T*_min_ = 0.835, *T*_max_ = 0.910	*k* = −1→20
4407 measured reflections	*l* = −14→13

### Refinement

**Table:**

Refinement on *F*^2^	Primary atom site location: structure-invariant direct methods
Least-squares matrix: full	Secondary atom site location: difference Fourier map
*R*[*F*^2^ > 2σ(*F*^2^)] = 0.063	Hydrogen site location: inferred from neighbouring sites
*wR*(*F*^2^) = 0.214	H-atom parameters constrained
*S* = 1.00	*w* = 1/[σ^2^(*F*_o_^2^) + (0.135*P*)^2^] where *P* = (*F*_o_^2^ + 2*F*_c_^2^)/3
3535 reflections	(Δ/σ)_max_ < 0.001
284 parameters	Δρ_max_ = 0.33 e Å^−^^3^
0 restraints	Δρ_min_ = −0.42 e Å^−^^3^

### Special details

**Table:**

Geometry. All e.s.d.'s (except the e.s.d. in the dihedral angle between two l.s. planes) are estimated using the full covariance matrix. The cell e.s.d.'s are taken into account individually in the estimation of e.s.d.'s in distances, angles and torsion angles; correlations between e.s.d.'s in cell parameters are only used when they are defined by crystal symmetry. An approximate (isotropic) treatment of cell e.s.d.'s is used for estimating e.s.d.'s involving l.s. planes.
Refinement. Refinement of *F*^2^ against ALL reflections. The weighted *R*-factor *wR* and goodness of fit *S* are based on *F*^2^, conventional *R*-factors *R* are based on *F*, with *F* set to zero for negative *F*^2^. The threshold expression of *F*^2^ > σ(*F*^2^) is used only for calculating *R*-factors(gt) *etc*. and is not relevant to the choice of reflections for refinement. *R*-factors based on *F*^2^ are statistically about twice as large as those based on *F*, and *R*- factors based on ALL data will be even larger.

### Fractional atomic coordinates and isotropic or equivalent isotropic displacement parameters (Å^2^)

**Table:**

	*x*	*y*	*z*	*U*_iso_*/*U*_eq_	
Mn1	1.0000	0.0000	1.0000	0.0507 (3)	
C1	0.8291 (4)	0.0451 (2)	0.7085 (3)	0.0579 (10)	
C2	0.8243 (4)	0.0985 (2)	0.7966 (4)	0.0572 (10)	
C3	0.7590 (4)	0.1738 (3)	0.7732 (4)	0.0670 (12)	
H3A	0.7183	0.1961	0.6991	0.080*	
C4	0.7671 (5)	0.2059 (3)	0.8768 (4)	0.0680 (12)	
H4A	0.7311	0.2541	0.8881	0.082*	
C5	0.8417 (4)	0.1524 (2)	0.9685 (4)	0.0572 (10)	
C6	0.8735 (4)	0.1665 (2)	1.0892 (4)	0.0570 (10)	
C7	0.9596 (4)	0.1198 (2)	1.1763 (4)	0.0551 (9)	
C8	1.0066 (4)	0.1385 (3)	1.2992 (4)	0.0617 (10)	
H8A	0.9820	0.1821	1.3345	0.074*	
C9	1.0931 (4)	0.0817 (3)	1.3555 (4)	0.0598 (10)	
H9A	1.1410	0.0798	1.4358	0.072*	
C10	1.0973 (4)	0.0248 (2)	1.2677 (3)	0.0542 (9)	
C11	0.7496 (4)	0.0648 (2)	0.5827 (4)	0.0594 (10)	
C12	0.8044 (5)	0.0722 (3)	0.4937 (4)	0.0697 (12)	
H12A	0.8948	0.0654	0.5115	0.084*	
C13	0.7273 (6)	0.0893 (3)	0.3793 (5)	0.0814 (14)	
H13A	0.7655	0.0920	0.3202	0.098*	
C14	0.5958 (6)	0.1024 (3)	0.3521 (5)	0.0864 (16)	
H14A	0.5448	0.1156	0.2752	0.104*	
C15	0.5383 (5)	0.0959 (3)	0.4383 (5)	0.0862 (15)	
H15A	0.4481	0.1041	0.4193	0.103*	
C16	0.6152 (5)	0.0771 (3)	0.5547 (4)	0.0701 (12)	
H16A	0.5761	0.0728	0.6129	0.084*	
C17	0.8100 (4)	0.2373 (2)	1.1233 (4)	0.0560 (10)	
C18	0.6787 (5)	0.2366 (3)	1.1115 (4)	0.0735 (12)	
H18A	0.6285	0.1915	1.0830	0.088*	
C19	0.6202 (5)	0.3030 (3)	1.1418 (5)	0.0822 (14)	
H19A	0.5314	0.3019	1.1346	0.099*	
C20	0.6936 (6)	0.3702 (3)	1.1824 (4)	0.0824 (15)	
H20A	0.6543	0.4145	1.2027	0.099*	
C21	0.8222 (6)	0.3720 (3)	1.1929 (5)	0.0829 (14)	
H21A	0.8712	0.4178	1.2197	0.099*	
C22	0.8817 (5)	0.3065 (3)	1.1642 (5)	0.0742 (13)	
H22A	0.9707	0.3084	1.1723	0.089*	
C23	1.2032 (10)	0.1503 (5)	1.0298 (13)	0.204 (6)	
H23A	1.1197	0.1743	1.0256	0.245*	
H23B	1.2537	0.1461	1.1133	0.245*	
C24	1.2665 (15)	0.2060 (6)	0.9868 (11)	0.227 (6)	
H24A	1.2841	0.2517	1.0374	0.341*	
H24B	1.2119	0.2210	0.9086	0.341*	
H24C	1.3481	0.1846	0.9839	0.341*	
P1	0.5000	0.0000	0.0000	0.1018 (9)	
N1	0.8781 (3)	0.0872 (2)	0.9162 (3)	0.0541 (8)	
N2	1.0169 (3)	0.05011 (19)	1.1572 (3)	0.0552 (8)	
F1	0.6259 (11)	0.0076 (5)	0.0792 (8)	0.217 (3)	
F2	0.4786 (14)	0.0803 (9)	−0.0209 (16)	0.350 (8)	
F3	0.460 (2)	0.0081 (15)	0.0848 (14)	0.387 (10)	
O1	1.1729 (3)	0.07288 (19)	0.9912 (3)	0.0787 (10)	

### Atomic displacement parameters (Å^2^)

**Table:**

	*U*^11^	*U*^22^	*U*^33^	*U*^12^	*U*^13^	*U*^23^
Mn1	0.0513 (5)	0.0471 (5)	0.0521 (5)	0.0032 (3)	0.0152 (4)	−0.0035 (3)
C1	0.062 (2)	0.057 (2)	0.052 (2)	0.0003 (19)	0.0165 (19)	−0.0015 (18)
C2	0.056 (2)	0.058 (2)	0.055 (2)	0.0046 (19)	0.0136 (18)	0.0003 (18)
C3	0.077 (3)	0.055 (2)	0.059 (2)	0.014 (2)	0.010 (2)	0.0028 (19)
C4	0.076 (3)	0.056 (2)	0.069 (3)	0.017 (2)	0.021 (2)	0.000 (2)
C5	0.057 (2)	0.054 (2)	0.060 (2)	0.0050 (18)	0.0173 (19)	−0.0075 (18)
C6	0.058 (2)	0.049 (2)	0.064 (2)	−0.0013 (18)	0.0195 (19)	−0.0075 (18)
C7	0.057 (2)	0.052 (2)	0.059 (2)	0.0011 (18)	0.0225 (19)	−0.0026 (18)
C8	0.073 (3)	0.054 (2)	0.060 (2)	0.003 (2)	0.024 (2)	−0.0111 (19)
C9	0.066 (3)	0.057 (2)	0.053 (2)	0.004 (2)	0.0163 (19)	−0.0026 (19)
C10	0.058 (2)	0.051 (2)	0.053 (2)	−0.0007 (19)	0.0185 (19)	−0.0005 (17)
C11	0.068 (3)	0.050 (2)	0.057 (2)	−0.0011 (19)	0.016 (2)	−0.0018 (18)
C12	0.071 (3)	0.071 (3)	0.065 (3)	−0.001 (2)	0.019 (2)	0.005 (2)
C13	0.098 (4)	0.078 (3)	0.066 (3)	0.001 (3)	0.026 (3)	0.010 (2)
C14	0.095 (4)	0.089 (4)	0.060 (3)	−0.005 (3)	0.005 (3)	0.008 (3)
C15	0.071 (3)	0.086 (4)	0.083 (3)	0.008 (3)	0.002 (3)	0.004 (3)
C16	0.062 (3)	0.078 (3)	0.065 (3)	0.003 (2)	0.014 (2)	0.002 (2)
C17	0.056 (2)	0.054 (2)	0.057 (2)	0.0087 (19)	0.0183 (18)	−0.0007 (18)
C18	0.067 (3)	0.061 (3)	0.092 (3)	0.004 (2)	0.026 (2)	−0.009 (2)
C19	0.073 (3)	0.083 (3)	0.098 (4)	0.019 (3)	0.038 (3)	0.000 (3)
C20	0.115 (5)	0.065 (3)	0.070 (3)	0.031 (3)	0.035 (3)	−0.004 (2)
C21	0.095 (4)	0.062 (3)	0.093 (4)	0.006 (3)	0.031 (3)	−0.015 (3)
C22	0.074 (3)	0.057 (3)	0.093 (3)	−0.004 (2)	0.030 (3)	−0.015 (2)
C23	0.187 (9)	0.105 (6)	0.404 (18)	−0.072 (6)	0.210 (11)	−0.095 (9)
C24	0.39 (2)	0.111 (7)	0.234 (13)	−0.052 (10)	0.170 (13)	−0.041 (7)
P1	0.0807 (16)	0.1047 (19)	0.1054 (19)	0.0316 (13)	0.0113 (14)	−0.0304 (15)
N1	0.0532 (18)	0.0542 (18)	0.0538 (18)	0.0027 (15)	0.0164 (15)	−0.0062 (14)
N2	0.0538 (19)	0.0549 (19)	0.0559 (18)	0.0009 (15)	0.0171 (15)	−0.0046 (15)
F1	0.216 (9)	0.238 (8)	0.206 (8)	−0.015 (6)	0.080 (7)	−0.014 (5)
F2	0.338 (16)	0.247 (13)	0.40 (2)	0.005 (12)	0.030 (15)	−0.013 (13)
F3	0.346 (19)	0.47 (2)	0.288 (18)	0.205 (16)	0.030 (14)	0.022 (15)
O1	0.0648 (19)	0.0609 (19)	0.116 (3)	−0.0143 (15)	0.0377 (19)	−0.0146 (18)

### Geometric parameters (Å, °)

**Table:**

Mn1—N1	2.004 (3)	C13—C14	1.358 (8)
Mn1—N1^i^	2.004 (3)	C13—H13A	0.9300
Mn1—N2	2.018 (3)	C14—C15	1.373 (8)
Mn1—N2^i^	2.018 (3)	C14—H14A	0.9300
Mn1—O1^i^	2.260 (3)	C15—C16	1.402 (7)
Mn1—O1	2.260 (3)	C15—H15A	0.9300
C1—C10^i^	1.395 (6)	C16—H16A	0.9300
C1—C2	1.403 (6)	C17—C18	1.371 (6)
C1—C11	1.504 (6)	C17—C22	1.394 (6)
C2—N1	1.369 (5)	C18—C19	1.390 (7)
C2—C3	1.432 (6)	C18—H18A	0.9300
C3—C4	1.331 (6)	C19—C20	1.375 (8)
C3—H3A	0.9300	C19—H19A	0.9300
C4—C5	1.442 (6)	C20—C21	1.346 (8)
C4—H4A	0.9300	C20—H20A	0.9300
C5—N1	1.386 (5)	C21—C22	1.377 (7)
C5—C6	1.393 (6)	C21—H21A	0.9300
C6—C7	1.388 (6)	C22—H22A	0.9300
C6—C17	1.497 (5)	C23—C24	1.358 (11)
C7—N2	1.381 (5)	C23—O1	1.388 (8)
C7—C8	1.425 (6)	C23—H23A	0.9700
C8—C9	1.348 (6)	C23—H23B	0.9700
C8—H8A	0.9300	C24—H24A	0.9600
C9—C10	1.436 (6)	C24—H24B	0.9600
C9—H9A	0.9300	C24—H24C	0.9600
C10—N2	1.387 (5)	P1—F3	1.23 (2)
C10—C1^i^	1.395 (6)	P1—F3^ii^	1.23 (2)
C11—C12	1.385 (6)	P1—F1^ii^	1.376 (11)
C11—C16	1.385 (6)	P1—F1	1.376 (11)
C12—C13	1.378 (7)	P1—F2	1.382 (15)
C12—H12A	0.9300	P1—F2^ii^	1.382 (15)
			
N1—Mn1—N1^i^	180.000 (1)	C14—C15—H15A	119.9
N1—Mn1—N2	90.13 (13)	C16—C15—H15A	119.9
N1^i^—Mn1—N2	89.87 (13)	C11—C16—C15	119.8 (5)
N1—Mn1—N2^i^	89.87 (13)	C11—C16—H16A	120.1
N1^i^—Mn1—N2^i^	90.13 (13)	C15—C16—H16A	120.1
N2—Mn1—N2^i^	180.000 (1)	C18—C17—C22	118.2 (4)
N1—Mn1—O1^i^	90.70 (13)	C18—C17—C6	120.8 (4)
N1^i^—Mn1—O1^i^	89.30 (13)	C22—C17—C6	121.0 (4)
N2—Mn1—O1^i^	90.25 (13)	C17—C18—C19	120.4 (5)
N2^i^—Mn1—O1^i^	89.75 (13)	C17—C18—H18A	119.8
N1—Mn1—O1	89.30 (13)	C19—C18—H18A	119.8
N1^i^—Mn1—O1	90.70 (13)	C20—C19—C18	120.0 (5)
N2—Mn1—O1	89.75 (13)	C20—C19—H19A	120.0
N2^i^—Mn1—O1	90.25 (13)	C18—C19—H19A	120.0
O1^i^—Mn1—O1	180.0	C21—C20—C19	120.2 (5)
C10^i^—C1—C2	123.3 (4)	C21—C20—H20A	119.9
C10^i^—C1—C11	119.2 (4)	C19—C20—H20A	119.9
C2—C1—C11	117.5 (4)	C20—C21—C22	120.4 (5)
N1—C2—C1	126.2 (4)	C20—C21—H21A	119.8
N1—C2—C3	109.7 (4)	C22—C21—H21A	119.8
C1—C2—C3	124.1 (4)	C21—C22—C17	120.8 (5)
C4—C3—C2	107.6 (4)	C21—C22—H22A	119.6
C4—C3—H3A	126.2	C17—C22—H22A	119.6
C2—C3—H3A	126.2	C24—C23—O1	128.0 (9)
C3—C4—C5	107.7 (4)	C24—C23—H23A	105.3
C3—C4—H4A	126.1	O1—C23—H23A	105.3
C5—C4—H4A	126.1	C24—C23—H23B	105.3
N1—C5—C6	126.7 (4)	O1—C23—H23B	105.3
N1—C5—C4	108.7 (3)	H23A—C23—H23B	106.0
C6—C5—C4	124.6 (4)	C23—C24—H24A	109.5
C7—C6—C5	123.8 (4)	C23—C24—H24B	109.5
C7—C6—C17	119.9 (4)	H24A—C24—H24B	109.5
C5—C6—C17	116.3 (4)	C23—C24—H24C	109.5
N2—C7—C6	125.6 (4)	H24A—C24—H24C	109.5
N2—C7—C8	109.6 (3)	H24B—C24—H24C	109.5
C6—C7—C8	124.8 (4)	F3—P1—F3^ii^	180 (2)
C9—C8—C7	108.0 (4)	F3—P1—F1^ii^	92.8 (9)
C9—C8—H8A	126.0	F3^ii^—P1—F1^ii^	87.2 (9)
C7—C8—H8A	126.0	F3—P1—F1	87.2 (9)
C8—C9—C10	107.0 (4)	F3^ii^—P1—F1	92.8 (9)
C8—C9—H9A	126.5	F1^ii^—P1—F1	180.0 (13)
C10—C9—H9A	126.5	F3—P1—F2	87.6 (9)
N2—C10—C1^i^	125.9 (4)	F3^ii^—P1—F2	92.4 (9)
N2—C10—C9	109.4 (4)	F1^ii^—P1—F2	84.2 (6)
C1^i^—C10—C9	124.7 (4)	F1—P1—F2	95.8 (6)
C12—C11—C16	118.4 (4)	F3—P1—F2^ii^	92.4 (9)
C12—C11—C1	123.1 (4)	F3^ii^—P1—F2^ii^	87.6 (9)
C16—C11—C1	118.4 (4)	F1^ii^—P1—F2^ii^	95.8 (6)
C13—C12—C11	121.1 (5)	F1—P1—F2^ii^	84.2 (6)
C13—C12—H12A	119.4	F2—P1—F2^ii^	180.0 (3)
C11—C12—H12A	119.4	C2—N1—C5	106.2 (3)
C14—C13—C12	120.4 (5)	C2—N1—Mn1	127.3 (3)
C14—C13—H13A	119.8	C5—N1—Mn1	126.3 (3)
C12—C13—H13A	119.8	C7—N2—C10	105.9 (3)
C13—C14—C15	120.0 (5)	C7—N2—Mn1	127.2 (3)
C13—C14—H14A	120.0	C10—N2—Mn1	126.7 (3)
C15—C14—H14A	120.0	C23—O1—Mn1	127.0 (4)
C14—C15—C16	120.2 (5)		

Symmetry codes: (i) −*x*+2, −*y*, −*z*+2; (ii) −*x*+1, −*y*, −*z*.
